# Tumor‐infiltrating immune cells predict the response to somatostatin receptor ligands only in somatotropinomas naïve to medical therapy

**DOI:** 10.1111/jne.70078

**Published:** 2025-08-11

**Authors:** Sabrina Chiloiro, Alessandra Vicari, Antonella Giampietro, Pier Paolo Mattogno, Natalia Cappoli, Greis Konini, Rosalinda Calandrelli, Liverana Lauretti, Simona Gaudino, Mario Rigante, Guido Rindi, Alessandro Olivi, Laura De Marinis, Antonio Bianchi, Francesco Doglietto, Marco Gessi, Alfredo Pontecorvi

**Affiliations:** ^1^ Dipartimento di Endocrinologia Fondazione Policlinico Universitario A. Gemelli, Istituto di Ricovero e Cura a Carattere Scientifico (IRCCS) Rome Italy; ^2^ Facoltà di Medicina e Chirurgia Università Cattolica del Sacro Cuore Rome Italy; ^3^ Dipartimento di Neurochirurgia Fondazione Policlinico Universitario A. Gemelli, Istituto di Ricovero e Cura a Carattere Scientifico (IRCCS) Rome Italy; ^4^ Unità di Anatomia Patologica, Dipartimento della donna e del bambino, e della salute pubblica Fondazione Policlinico Universitario A. Gemelli, Istituto di Ricovero e Cura a Carattere Scientifico (IRCCS) Rome Italy; ^5^ Dipartimento di Diagnostica per Immagini e Radioterapia Oncologica Fondazione Policlinico Universitario A. Gemelli, Istituto di Ricovero e Cura a Carattere Scientifico (IRCCS) Rome Italy; ^6^ Unità di Otolarinolaringoiatria Fondazione Policlinico Universitario A. Gemelli, Istituto di Ricovero e Cura a Carattere Scientifico (IRCCS) Rome Italy

**Keywords:** acromegaly, lymphocytes, macrophages, microenvironment, pituitary adenoma

## Abstract

Tumor‐infiltrating immune cells (TICs) are important components of the tumor microenvironment (TME). They regulate somatotroph adenoma treatment responses to therapy with somatostatin receptor ligands (SRLs), mediated by soluble factors and cytokines. In this study, we assessed the effect of SRLs treatment on TICs. A retrospective and observational study was performed on acromegaly patients to compare the number of TICs in 75 patients naïve to SRL before surgery and in 33 patients treated with SRL for at least 6 months before surgery. In SRLs‐naive patients at surgery, the CD68+/CD8+ ratio was higher in invasive tumors (4.9, IQR: 14, *p* = .028) than in non‐invasive tumors (4.3, IQR: 4.2) as well as in patients not responsive to post‐surgical/adjuvant treatment with SRLs (7.5, IQR: 13, *p* = .006) than those responsive to treatment (3.4, IQR: 3.2). In patients treated with SRLs before surgery, the number of CD68+ macrophages and the ratio CD68+/CD8+ were lower in patients non‐responsive to post‐surgery/adjuvant SRL treatment (CD68+: 48/HPFs, IQR: 22.9, *p* = .005; CD68+/CD8+: 2.0, IQR: 3.6, *p* = .05) than in responsive patients (CD68+: 80/HFPs, IQR: 51, CD68+/CD8+: 5, IQR: 5.6). Higher CD68+/CD8+ ratio was an independent risk factor for post‐surgery SRL treatment resistance, only in patients naïve to SRLs at surgery (OR: 4.3, 95% IC: 1.4–12.9, *p* = .006). Our results indicate a presurgical SRL therapy interplay with TICs in somatotroph adenomas and show that the CD68+/CD8+ ratio is a biomarker for treatment resistance in SRL‐naïve patients.

**Clinical Trial Registration:**

The Clinical Trial Registration number is 5116.

## 1 INTRODUCTION

The tumoral microenvironment (TME) represents an interplay between tumor and non‐tumorous cells and includes immune cells, soluble factors, cytokines, blood and lymphatic vessels, fibroblasts, and extracellular matrix components, generating a complex inter‐cellular network.^1^ The TME may be predictive of response to medical therapies in pituitary adenomas.^2^

Immune cells play an important role in the TME by mediating complex interactions, many of which remain yet unknown.^3^ The phenotype and functional activities of both tumor‐infiltrating lymphocytes (TILs) and tumor‐associated macrophages (TAMs) are complex. Some TILs behave as effector cells, such as CD8+ and natural killer lymphocytes,^4^ inducing a cytotoxic cascade resulting in tumor cell death, while other TILs show a regulatory role inhibiting the anti‐tumor activity of effector T cells, mainly CD4+ lymphocytes.^5^ Similarly, CD68+ macrophages can be differentiated into M1 and M2 macrophages, with TAMs generally thought to resemble M2‐polarized macrophages, which play a role in promoting tumor cell proliferation and progression as well as inhibiting immune response mediated by T lymphocytes.^6^ CD138, which belongs to the syndecan family, is predominantly expressed in epithelial cells and plasmacytes.^7^ It is the defining marker that enables identification of human plasma cells.^8^ CD138 also plays a role in molecular pathways involved in cell proliferation, apoptosis, angiogenesis, tumor invasion, and metastasis.^7^ TME participates in regulating neo‐angiogenesis, tumor invasive growth, and response to treatments, and also serves as a target for medical therapies, such as immune checkpoint inhibitors (ICIs), monoclonal antibodies directed against the epithelial growth factors (EGF) and against vascular endothelial growth factor (VEGF).^9^

Tumors infiltrating immune cells differ according to subtypes of pituitary adenomas.^10^ In pituitary transcription factor 1 (PIT1) lineage‐derived pituitary somatotroph, lactotroph, mammosomatotroph, and thyrotrope tumors, infiltrating immune cells are mainly CD8+ lymphocytes and M2‐polarized macrophages.^11^ Pituitary adenomas were recently classified according to types and number of infiltrating immune cells and to the expression of checkpoint molecules, such as the cytotoxic T‐lymphocyte antigen‐4 (CTLA‐4), the programmed cell death protein‐1 (PD‐1) and the programmed cell death protein ligand‐2 (PD‐L2).^10^ The role of infiltrating immune cells in somatotropinomas has been partially investigated. Fewer tumor‐infiltrating CD8+ lymphocytes were found in tumors with cavernous sinus invasion and in those non‐responsive to post‐surgical somatostatin receptor ligand (SRL) treatment. A high CD68+/CD8+ ratio was also observed.^12^, ^13^ The effects of medical therapies on TME are largely unknown.^14^ As shown for other endocrine tumors, tumor‐associated fibroblasts (TAFs),^15^ B cells, and macrophages express somatostatin receptors (SSTRs).^16^ In acromegaly, presurgical therapy with SRLs remains controversial, as reported in current guidelines.^17^, ^18^ However, the effects of SRLs on biology, molecular profile (such as proliferative index, SSTR expression) and tumor infiltrating immune cells have not been extensively investigated or comprehensively clarified in somatotropinomas.

In this study, we aim to investigate the number of somatotroph adenoma infiltrating immune cells (CD68+ macrophages, CD138+ and CD8+ lymphocytes) in acromegaly patients naïve to medical therapy before surgery and in patients pretreated with SRL before surgery.

## 2 PATIENTS AND METHODS

A retrospective, observational, monocenter cohort study was performed on patients with acromegaly diagnosed and followed at the Pituitary Unit and at the Department of Neurosurgery of Gemelli University Hospital between 2012 and 2022.

### 2.1 Objectives

The primary objective of this study was to investigate the number of somatotroph adenoma infiltrating immune cells (CD68+ macrophages, CD138+ and CD8+ lymphocytes) in acromegaly patients naïve to medical therapy with SRL before surgery and in patients treated with SRL for at least 6 months before surgery.

As secondary objectives, we investigated the predictors of responsiveness to post‐surgical therapy with SRLs in these two cohorts.

### 2.2 Inclusion/exclusion criteria

Patients were consecutively included in the study according to the following inclusion/exclusion criteria.

Inclusion criteria were:

1. Clinical and biochemical diagnosis of acromegaly;
2. Histology diagnosis of pure somatotroph adenomas, with positive immunohistochemistry for GH and Pit‐1^19^;
3. Available formalin‐fixed and paraffin‐embedded somatotroph adenoma specimens for experimental analysis;
4. Agreement to participate in the study by signing an informed consent.

Exclusion criteria were:

1. Medical treatment with dopamine agonist before pituitary surgery;
2. Histology diagnosis of mixed somatotroph tumor, co‐expressing GH with other pituitary hormones^19^;
3. Pituitary apoplexy;
4. Head and neck radiotherapy within 10 years before pituitary surgery;
5. Previous or concomitant treatment with glucocorticoids;
6. Previous or active immune‐related disease of other organs or tissues.

### 2.3 Data collection

Demographic, clinical, hormonal, molecular, and tumor morphology data were collected from patient medical records.

The following information was included: gender, age, growth hormone (GH) and insulin‐like growth factor‐I (IGF‐I) levels at acromegaly diagnosis, tumor morphology (tumor maximum diameter and pattern of growth/invasion), tumor proliferative index (MIB‐1), expression of the subtype 2a of the somatostatin receptor expression (SSTR2) at immunohistochemistry, surgical outcome, and response to treatment with first generation SRL.

Invasive adenomas were defined with infiltration of the sellar floor (involving inferiorly the sphenoid sinus and nasopharynx), the lateral margin of the cavernous sinus, the arachnoid, and the posterior fossa.^20^ Cavernous sinus invasion was defined radiologically as a Knosp grade of 3 or 4.^21^ Cavernous sinus invasion had also to be confirmed intraoperatively.^22^, ^23^

Surgery outcome was defined according to IGF‐I levels measured at 3 and 6 months after surgery, both in patients naïve to SRL treatment and in patients treated with SRLs before surgery, according to recent consensus criteria for acromegaly diagnosis and remission.^24^ The presence of post‐operative macroscopic residual of somatotroph tumor was defined according to the pituitary magnetic resonance imaging (MRI) performed 3–6 months after surgery.^24^ Patients affected by active acromegaly after surgery were treated with SRLs with a conventional dose (Lanreotide Atg 120 mg or Octreotide Lar 30 mg every 28 days). SRL responses were defined according to the recent literature, after at least 6 consecutive months of standard dose therapy^25^, ^26^ SRL responsiveness was defined in patients who reached IGF‐I levels within the normal ranges adjusted for age. Patients not responsive to SRLs were switched to other medical therapies (Pasireotide Lar or Pegvisomant).

### 2.4 Histopathological assessment

Histopathological assessment was conducted according to our clinical practice on somatotroph adenoma samples of all the patients included in the study. Specimens were examined for pituitary hormones and transcription factors, pituitary‐specific transcription factor (Pit‐1), GATA binding protein 3 (GATA‐3); proliferative index (MIB‐1), cytokeratin (CAM5.2) and TME components (Table S1). The MIB‐1 index was expressed as a percent of positive nuclei in “hot spot” areas.^22^

Somatotroph adenomas were classified as sparsely granulated, densely granulated, or intermediate phenotype based on morphology and cytokeratin pattern.^27^ Briefly, fibrous bodies in >70% of adenoma cells, irrespective of the percentage of transitional cells, defined sparsely granulated somatotroph adenomas. Densely granulated somatotroph adenomas showed perinuclear cytokeratin in >70% of cells or fibrous bodies in <8%, irrespective of the percentage of transitional pattern cells. Adenomas that could not be classified as densely or sparsely granulated were defined as intermediate type. Intermediate type adenomas were grouped with densely granulated somatotroph adenomas, as suggested.^27^

The number of clusters of differentiation (CD)‐68+ macrophages, CD138+ and CD8+ lymphocytes was expressed as the total number of positive cells in four high‐power fields (1 HPF: 0.25 mm^2^).^12^, ^28^ Fields were randomly selected within somatotroph tumor tissue, avoiding areas close to vessels or in areas of hemorrhage. Positive cells of four sequential fields were counted. Cells were considered positive only if the cellular nucleus was identified. Fields were randomly selected within somatotroph tumor tissue. Fields were not analyzed if sited close to vessels or in areas of doubt, such as the interface between the tumor and the normal pituitary gland. Appropriate positive control slides with immunohistochemistry for CD45 cells were included for each staining, while one section was processed with omission of the primary antibody as a negative control. Cells in four sequential fields were counted and considered positive only if the cellular nucleus was identified.

### 2.5 Statistical analysis

Descriptive statistics were used to report the clinical and demographic characteristics of the patient cohort. The normality of continuous variables was tested using the Kolmogorov–Smirnov test. Quantitative variables were expressed as median and interquartile range (IQR) and qualitative variables as absolute and percentage frequencies. To investigate the difference of tumor infiltrating immune cells (TICs) among patients naïve to fg‐SRLs treatment and among patients treated with fg‐SRLs before surgery, the chi‐squared test (or Fisher exact test when appropriate) and Mann–Whitney non‐parametric tests were used to compare categorical and quantitative unpaired data. All variables were subjected to univariable logistic analysis, and covariates found to be associated (exploratory univariate *p* < .05) with rejection were entered into a multivariable logistic regression model as independent covariates. A stepwise selection method (*p* < .05) and the Ridge regression were applied to identify the final regression model. Analyses were performed with SPSS software version 30.0 for Windows.

## 3 RESULTS

One hundred and eight patients were included in the study. Clinical, morphological, and pathological findings of the study cohort are depicted in Table 1. Sixty‐five patients were female (60.2%), and 43 patients were male (39.8%). The median age at acromegaly diagnosis was 47 years (IQR: 20). Median GH at acromegaly diagnosis was 9.3 ng/mL (IQR: 15.2), median IGF‐I × ULN at acromegaly diagnosis was 2.6 (IQR: 1.3) and median prolactin was 7.9 ng/mL (IQR: 7.7). Ninety‐seven patients had a macroadenoma (89.8%), and 11 patients had a microadenoma (10.2%) The median maximum diameter was 15 mm (IQR: 6). Invasive tumor growth was observed in 36 patients (33.3%).

**TABLE 1:** Clinical, biochemical, morphological, and pathological features of the whole study cohort, and patients naïve to fg‐SRLs therapy at surgery versus patients treated with fg‐SRLs before surgery.

	Whole cohort	Naïve to fg‐SRLs therapy patients at surgery	Pre‐surgery fg‐SRLs treated patients	*p*‐value
Gender				
Females *n*, (%)	65 (60.2%)	50 (66.7%)	15 (45.5%)	.04
Males *n*, (%)	43 (39.8%)	25 (33.3%)	18 (54.5%)	
Age at acromegaly diagnosis	47 (20)	45 (20)	51 (15)	.108
GH at acromegaly diagnosis ng/mL, median IQR	9.3 (15.2)	9.4 (14.4)	9.1 (15.8)	.47
IGF‐I × ULN at acromegaly diagnosis, median IQR	2.6 (1.3)	2.5 (1.3)	2.9 (2)	.314
PRL at acromegaly diagnosis ng/mL, median IQR	7.9 (7.7)	8 (18)	7.9 (5.1)	.062
Tumor dimension				
Microadenoma *n*, (%)	11 (10.2%)	4 (5.3%)	7 (21.2%)	.012
Macroadenoma *n*, (%)	97 (89.8%)	71 (73.2%)	26 (78.8%)	
Maximum diameter mm median IQR	15 (6)	15 (6)	12.5 (7)	.272
Pattern of growth				
Not invasive tumors *n*, (%)	72 (66.7%)	51 (68%)	21 (63.6%)	.658
Invasive tumors *n*, (%)	36 (33.3%)	24 (32%)	12 (36.4%)	
Cytokeratin pattern				
Densely/intermediate granulated *n*, (%)	42 (42.9%)	30 (41.7%)	12 (46.2%)	.692
Sparsely granulated *n*, (%)	56 (57.1%)	42 (58.3%)	14 (53.8%)	
SSTR2a expression				
Absent/cytoplasmic immunoreactivity *n*, (%)	34 (32.7%)	20 (27.8%)	14 (43.8%)	.109
Membranous staining *n*, (%)	70 (67.3%)	52 (72.2%)	18 (56.2%)	
Proliferative index				
MIB1 <3%	66 (63.5%)	39 (54.9%)	27 (81.8%)	.008
MIB1 >3%	38 (36.5%)	32 (45.1%)	6 (18.2%)	
Surgery outcome				
Complete/gross total resection *n*, (%)	62 (57.4%)	38 (50.7%)	24 (72.7%)	.033
Partial resection *n*, (%)	46 (42.6%)	37 (49.3%)	9 (27.3%)	
Outcome of adjuvant fg‐SRLs therapy				
Response *n*, (%)	45 (57.7%)	36 (57.1%)	9 (60%)	.84
Not‐response *n*, (%)	33 (42.3%)	27 (42.9%)	6 (40%)	
CD8+ lymphocytes median (IQR)	13 (16.3)	10.6 (19)	17 (12.7)	.27
CD68+ macrophages median (IQR)	56 (40.8)	55 (41)	62 (39.7)	.81
CD138+ lymphocytes median (IQR)	4 (6)	6 (8.7)	3 (6)	.223
CD68+/CD8+ ratio median (IQR)	4.4 (6.4)	4.4 (5.5)	4.3 (6.6)	.27

*Note*: Univariate analysis.

Thirty‐three patients (30.6%) were treated with SRLs for 6 months before surgery: 10 with octreotide Lar 30 mg/monthly and 23 with Lanreotide Atg 120 mg/monthly. The remaining 75 patients were naïve to medical therapies at neurosurgery (69.4%). Pre‐surgery treatment with SRLs was prescribed according to the clinical judgment, mainly in patients with acromegaly‐related comorbidities, such as obstructive sleep apnea syndrome.

Twenty patients were reported in our previous studies that were designed with the different aim of investigating the prognostic role of tumor infiltrating immune cells.^12^, ^28^

A gross‐total resection was achieved in 62 (57.4%) and a partial resection in 46 patients (42.6%). Seventy‐eight patients were treated with SRLs also after adenoma resection. Forty‐five patients were considered good responders (57.7%), and 33 patients were considered not responsive (42.3%).

### 3.1 Clinical assessment of patients naïve to medical therapy and patients who underwent presurgical SRL treatment

Clinical, morphological, and pathological findings in patients naïve to medical therapy and patients who underwent presurgical SRL treatment are depicted in Table 1. Patients naïve to medical therapy at surgery were more frequently females (50 out of 75, 66.7%), while patients treated with SRLs before surgery were more frequently males (18 out of 33, 55.5%, *p* = .04), as reported in Table 1. Age, GH, IGF‐I, and PRL levels at acromegaly diagnosis were similar between patients naïve to SRL therapy at surgery and patients treated with SRLs before surgery (Table 1). Patients naïve to SRLs had more frequent macroadenomas (71 of 97, 73.2%). In contrast, patients treated with SRLs before surgery had more frequent microadenomas (7 of 11, 63.3%, *p* = .012). Adenoma growth patterns (invasive or non‐invasive) did not differ among patients naïve to presurgical SRLs and patients treated with SRLs before surgery (Table 1).

Complete/gross total resection was reached more frequently in patients treated with SRLs before surgery (24 out of 62, 72.7%) than in those naïve to SRL therapy at surgery (38 out of 62, 50.7%, *p* = .033).

The efficacy of post‐surgical SRL treatment did not differ between patients treated with SRLs before surgery and patients naïve to SRLs therapy at surgery.

### 3.2 Pathological assessment

All patients were diagnosed with somatotroph adenomas. The MIB‐1 index was <3% in 66 patients (63.5%) and higher than 3% in 38 specimens (36.5%). Forty‐two adenomas were densely granulated (42.9%), and 56 were sparsely granulated (57.1%). Absent or cytoplasmic SSTR2A expression was detected in 34 specimens (32.7%) and SSTR2a membranous staining was detected in 70 specimens (67.3%). The number of tumor infiltrating CD8+ lymphocytes was 13/HPFs (IQR: 16.3), of CD68+ macrophages was 56/HPFs (IQR: 40.8), and of CD138+ lymphocytes was 4/HPFs (IQR: 6). The CD68+/CD8+ immune cell ratio was 4.4 (IQR: 6.4). The number of tumor infiltrating CD8+ and CD138+ lymphocytes, and of CD68+ macrophages, and the CD68+/CD8+ ratio did not differ according to clinical, biochemical, pathological, and morphological features of the study population (Table 2).

**TABLE 2:** Tumor infiltrating CD8+, CD138+, CD68+ cells and CD68+/CD8+ ratio according to clinical, biochemical, morphological, and pathological features of the whole study cohort.

	CD8+ lymphocytes, median (IQR)	*p*	CD138+ lymphocytes, median (IQR)	*p*	CD68+ macrophages, median (IQR)	*p*	CD68+/CD8+ ratio, median (IQR)	*p*
Gender		.378		.071		.377		.78
Females	11 (18)		6 (5)		56 (42)		4.6 (6.5)	
Males	17 (16)		2.5 (6.7)		64 (42)		3.9 (6.4)	
Age at acromegaly diagnosis	n.a	.228	n.a	.895	n.a	.171	n.a	.347
GH at acromegaly diagnosis	n.a	.465	n.a	.939	n.a	.337	n.a	.087
IGF‐I at acromegaly diagnosis	n.a	.946	n.a	.521	n.a	.255	n.a	.594
PRL at acromegaly diagnosis	n.a	.189	n.a	.734	n.a	.111	n.a	.425
Tumor dimension		.241		.351		.655		.262
Microadenoma	17 (10)		3 (6)		68 (48)		4 (2.8)	
Macroadenoma	12 (17)		4 (6)		57 (43)		4.4 (6.7)	
Maximum diameter	n.a	.752	n.a	.112	n.a	.742	n.a	.816
Pattern of growth		.208		.338		.963		.759
Not invasive tumors	13 (17.7)		6 (9.2)		56 (47)		4.4 (3.9)	
Invasive tumors	10.5 (17.8)		3.5 (4.7)		62 (36)		4.9 (13)	
Cytokeratin pattern		.336		.302		.853		.105
Densely/intermediate granulated	14.4 (14.5)		3 (7)		64 (33)		3.4 (4.1)	
Sparsely granulated	10.8 (17.8)		6 (8)		55 (47)		5.4 (7.8)	
SSTR2a expression		.956		.513		.043		.361
Absent/cytoplasmic immunoreactivity	13 (19.1)		3.5 (6.5)		65 (47)		4.9 (10)	
Membranous staining	13 (15.2)		6 (8.5)		54 (38)		4.4 (5.5)	
Proliferative index		.589		.195		.918		.428
MIB1 <3%	13 (17.2)		4 (7)		57 (38)		3.9 (6.4)	
MIB1 >3%	11.5 (16.4)		6 (36)		55 (51)		5.5 (6.5)	
Surgery outcome		.102		.77		.501		.788
Complete/gross total resection	14 (18)		5 (7)		57 (41)		4.4 (4.3)	
Partial resection	10.3 (17.8)		4.5 (8.7)		60 (43)		5 (7)	

*Note*: Univariate analysis.

Abbreviation: n.a, not applicable.

The proliferative index (MIB‐1) was more frequently <3% in patients treated with SRLs before surgery (27/66, 81.8%) than in patients naïve to SRLs at surgery (36/66, 54.9%, *p* = .008), as shown in Table 1.

As we previously reported, the number of CD8+ lymphocytes and the ratio CD68+/CD8+ immune cells correlated with invasive growth^13^ and with the post‐surgical response to SRLs.^12^, ^28^ We investigated the tumor immune profile in patients naïve to SRLs at surgery and in patients who underwent presurgical treatment with SRLs, according to invasive growth (invasive and not‐invasive tumors) and according to the outcome of post‐surgery SRL outcome (responsive and non‐responsive patients).

**FIGURE 1:**
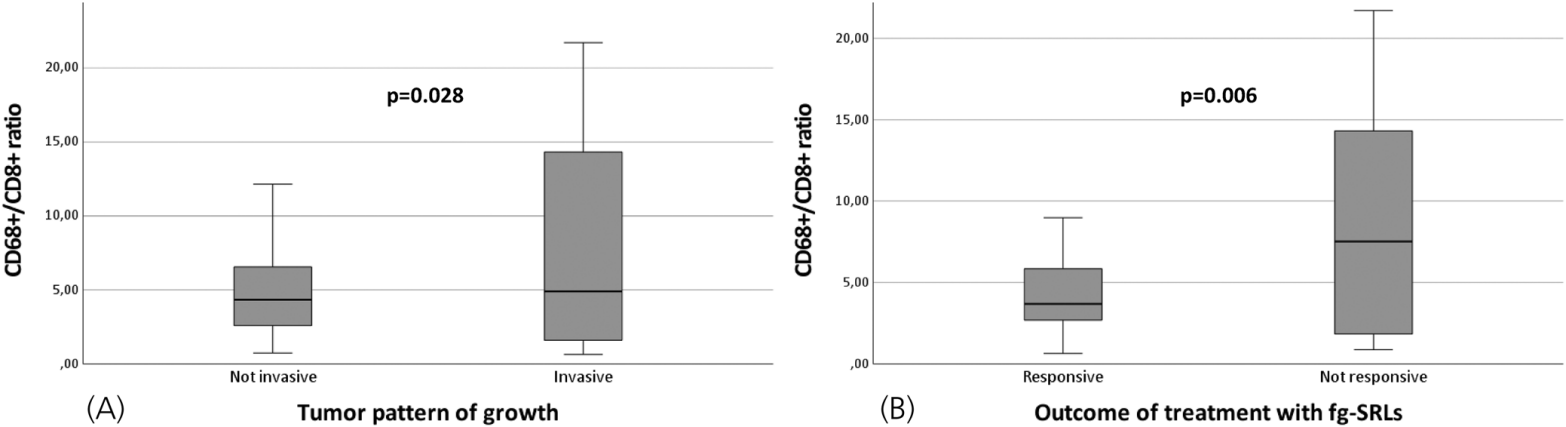
Box‐plot representing CD68+/CD8+ ratio according to tumor pattern growth (A) and outcome of post‐surgery treatment with fg‐SRLs among patients naïve to treatment with fg‐SRLs before surgery (B).

**FIGURE 2:**
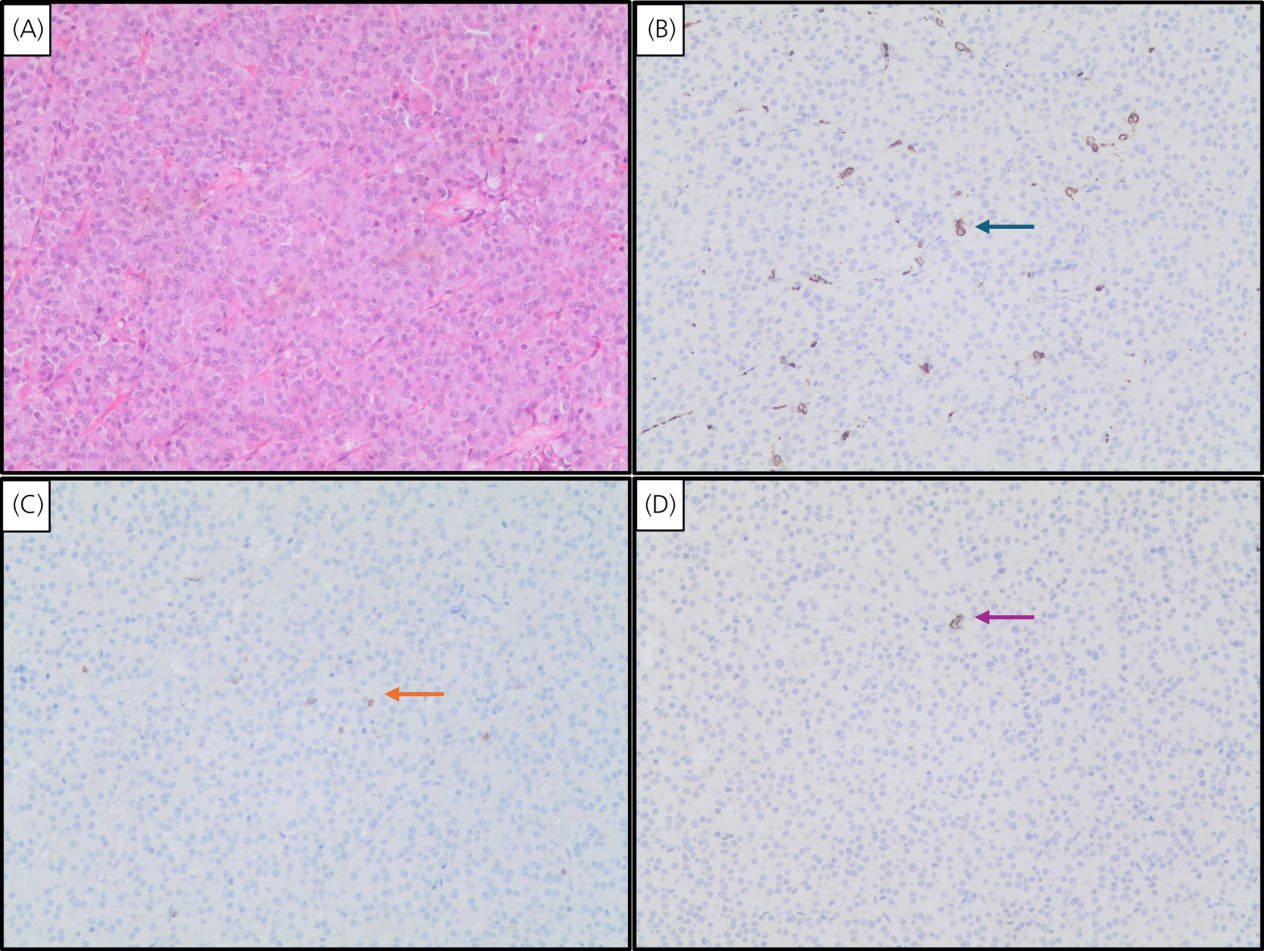
Representative immunohistochemistry pictures for H&E staining (A); CD68+ macrophages (B) that are indicated with a green arrow; CD8+ lymphocytes (C) that are indicated with an orange arrow; and CD138+ lymphocytes (D) that are indicated with a pink arrow. Magnification 150× in a sample of a somatotroph tumor naïve to treatment with fg‐SRLs before surgery.

In patients naïve to fg‐SRLs at surgery, the CD68+/CD8+ ratio was higher in invasive tumors (4.9, IQR: 14, *p* = .028) than in non‐invasive tumors (4.3, IQR: 4.2) and in patients not responsive to post‐surgical/adjuvant SRL treatment (7.5, IQR: 13, *p* = .006) than in those responsive to post‐surgical/adjuvant SRL treatment (3.4, IQR: 3.2), as shown in Figure 1 and Table 3. Representative images of CD68+, CD8+, and CD138+ immune cells in a somatotroph adenoma sample naïve to SRLs at surgery are shown in Figure 2. No significant differences were detected in the number of tumor‐infiltrating CD68+ macrophages, CD8+, and CD138+ lymphocytes among patients naïve to SRL at surgery and responsive to post‐surgery SRL treatment and patients naïve to SRL at surgery and not responsive to post‐surgery SRLs, as reported in Table 3.

**TABLE 3:** Predictors of response to post‐surgery treatment with fg‐SRLs in patients naïve to fg‐SRLs therapy at surgery versus patients treated with fg‐SRLs before surgery.

	Naïve to SRLs therapy patients at surgery			Pre‐surgery SRLs treated patients		
	Outcome of post‐surgery fg‐SRLs			Outcome of post‐surgery fg‐SRLs		
	Responsive	Not‐responsive	*p*‐value	Responsive	Not‐responsive	*p*‐value
Gender			.446			.205
Females *n*, (%)	22 (61.1%)	19 (70.4%)		3 (33.3%)	4 (66.7%)	
Males *n*, (%)	14 (38.9%)	8 (29.6%)		6 (66.7%)	2 (33.3%)	
Age at acromegaly diagnosis	47.5 (18.7)	38 (16)	.2	47 (16.5)	36 (31.7)	.529
GH at acromegaly diagnosis ng/mL, median IQR	8.7 (16.4)	11.6 (10.5)	.361	10.4 (32.3)	6.9 (43)	.886
IGF‐I × ULN at acromegaly diagnosis, median IQR	2.6 (1.3)	2.1 (1.3)	.725	3.4 (2.8)	1.9 (1)	.177
PRL at acromegaly diagnosis ng/mL, median IQR	7 (4.5)	19.2 (37.8)	.073	8.2 (5.7)	15 (16)	.533
Tumor dimension			.383			.215
Microadenoma *n*, (%)	1 (2.8%)	0 (0%)		2 (22.2%)	0 (0%)	
Macroadenoma *n*, (%)	35 (97.2%)	27 (100%)		7 (77.8%)	6 (100%)	
Maximum diameter mm median IQR	15 (6)	16 (9.2)	.836	14 (15.2)	30 (20)	.267
Pattern of growth			.001			.08
Not invasive tumors *n*, (%)	31 (86.1%)	10 (37%)		6 (66.7%)	1 (16.7%)	
Invasive tumors *n*, (%)	5 (13.9%)	17 (63%)		3 (33.3%)	5 (83.3%)	
Cytokeratin pattern	20 (55.6%)	7 (25.9%)	.02	6 (66.7%)	2 (40%)	.334
Densely/intermediate granulated *n*, (%)	16 (44.4%)	20 (74.1%)		3 (33.3%)	3 (60%)	
Sparsely granulated *n*, (%)						
SSTR2a expression			.015			.143
Absent/cytoplasmic immunoreactivity *n*, (%)	5 (13.9%)	11 (40.7%)		1 (11.1%)	3 (50%)	
Membranous staining *n*, (%)	31 (86.1%)	16 (59.3%)		8 (88.9%)	3 (50%)	
Proliferative index			.035			.025
MIB1 <3%	23 (63.9%)	10 (37%)		8 (88.9%)	2 (40%)	
MIB1 >3%	13 (36.1%)	17 (63%)		1 (11.1%)	3 (60%)	
Surgery outcome			.001			.08
Complete/gross total resection *n*, (%)	21 (58.3%)	5 (18.5%)		6 (66.7%)	1 (16.7%)	
Partial resection *n*, (%)	15 (41.7%)	22 (81.5%)		3 (33.3%)	5 (83.3%)	
CD8+ lymphocytes median (IQR)	14.5 (17.5)	9.3 (15.7)	.143	18 (8)	20.3 (39)	.328
CD68+ macrophages median (IQR)	52.4 (36.4)	61.2 (53)	.402	80 (51)	48 (22.9)	.005
CD138+ lymphocytes median (IQR)	6 (8)	5 (14)	.998	1 (4)	3 (22)	.222
CD68+/CD8+ ratio median (IQR)	4.1 (3.4)	6.1 (11.1)	.033	5 (5.6)	2 (3.6)	.05

*Note*: Univariate analysis.

**FIGURE 3:**
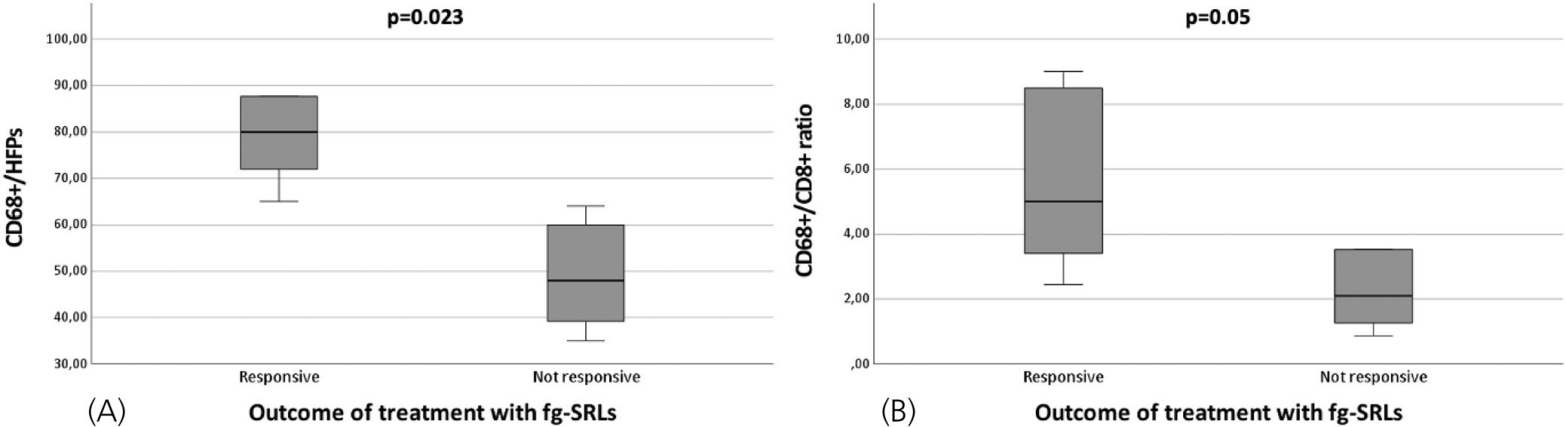
Box‐plot representing CD68+/HPFs (A) and CD68+/CD8+ ratio (B) according to the outcome of post‐surgery treatment with fg‐SRLs among patients treated with fg‐SRLs before surgery.

**FIGURE 4:**
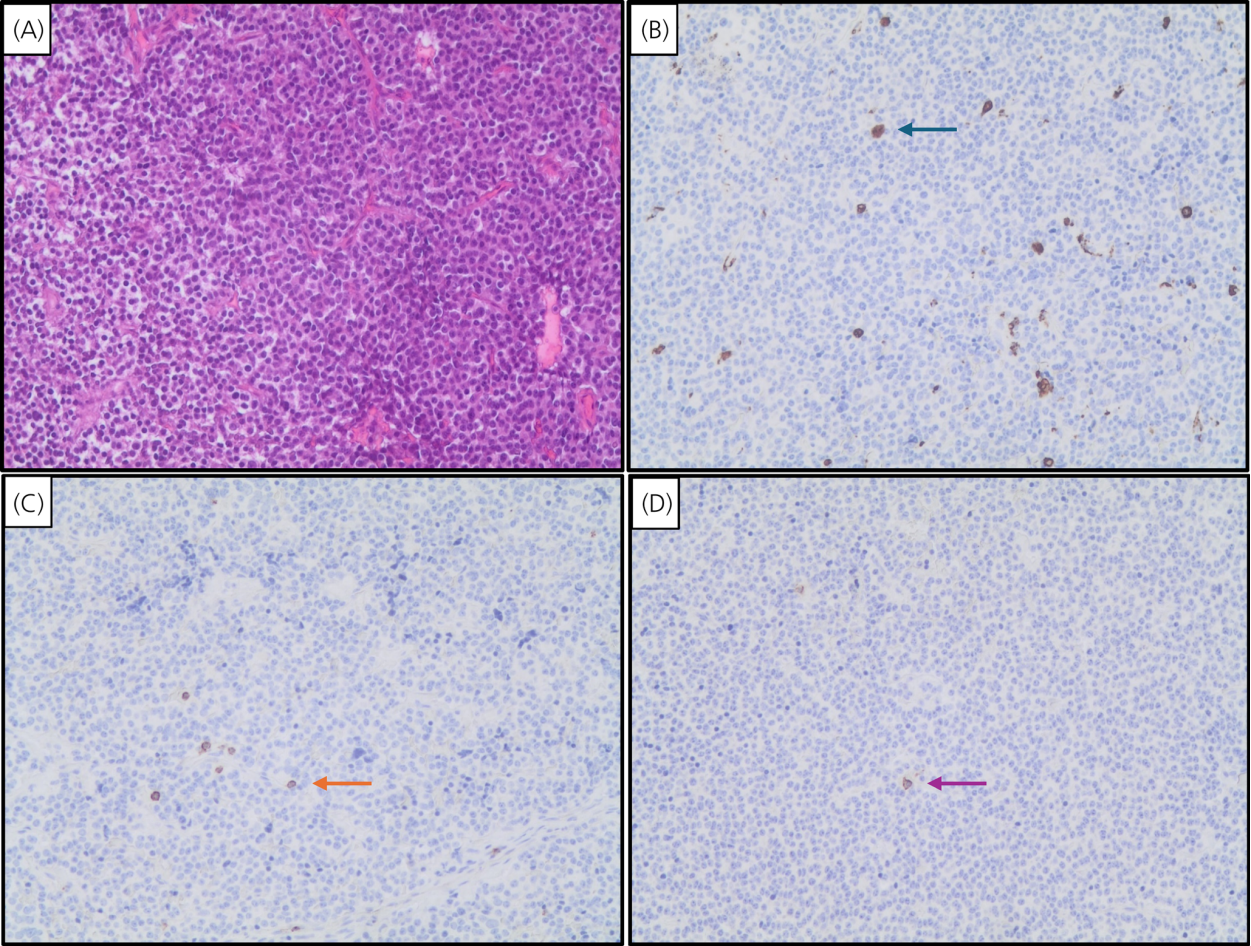
Representative immunohistochemistry pictures for H&E staining (A); CD68+ macrophages (B) that are indicated with a green arrow; CD8+ lymphocytes (C) that are indicated with an orange arrow; and CD138+ lymphocytes (D) that are indicated with a pink arrow. Magnification 150× in a sample of a somatotroph tumor treated with fg‐SRLs before surgery.

In patients treated with SRLs before surgery, the number of tumor infiltrating CD8+ and CD138+ lymphocytes, CD68+ macrophages, and the CD68+/CD8+ ratio did not differ among invasive and non‐invasive tumors. However, the number of CD68+ macrophages and the ratio of CD68+/CD8+ were lower in patients not responsive to post‐surgery/adjuvant SRL treatment (median CD68+: 48/HPFs, IQR: 22.9, *p* = .005; median CD68+/CD8+: 2.0, IQR: 3.6, *p* = .05) than in responsive patients (median CD68+ cell: 80/HFPs, IQR: 51; median CD68+/CD8+: 5, IQR: 5.6), as shown in Figure 3 and Table 3. Representative images of CD68+, CD8+, and CD138+ immune cells in a somatotroph adenoma sample treated with SRLs before surgery are shown in Figure 4. No significant differences were detected with regard to CD8+ and CD138+ lymphocytes.

### 3.3 Predictors of response to post‐surgical SRL treatment in patients naïve to SRL treatment

Resistance to post‐surgical SRL treatment in patients naïve to SRL treatment at surgery was more frequent in invasive (17 out of 27, 63%) than in non‐invasive adenomas (10 out of 27, 37%, *p* < .001), as shown in Table 3. In SRL‐naïve patients, a complete/gross total resection was more frequently achieved in patients responsive to post‐surgical SRLs (21 of 36, 58.3%) than in patients non‐responsive to post‐surgical SRLs (5 of 27, 18.5%, *p* < .001). In this group, a response to post‐surgical SRLs occurred more frequently in adenomas with a densely granulated cytokeratin pattern (20 of 36 specimens, 55.6%) than in adenomas with a sparsely granulated cytokeratin pattern (16 of 36 specimens, *p* = .02). Furthermore, the response to post‐surgical SRLs was more frequent in adenomas with SSTR2a membranous staining (31 of 36 cases, 86.1%) than in those with absent/cytoplasmic SSTR2a expression (5 of 36 specimens 13.9%, *p* = .015). It was also more frequent in those with a proliferative index <3% (23 of 36 specimens, 63.9%) than in those with a proliferative index >3% (13 of 36 cases, 36.1%, *p* = .035). Moreover, the CD68+/CD8+ ratio was lower in patients responsive to post‐surgical SRL treatment (4.1, IQR: 3.4) than in non‐responsive patients (6.1, IQR: 11.1, *p* = .033), as shown in Table 3.

### 3.4 Predictors of response to post‐surgical SRL treatment in patients treated before surgery with SRLs

In patients who underwent presurgical SRL therapy, a proliferative index <3% was more frequently associated with a higher frequency of post‐surgical SRL responsiveness (8 of 9, 88.9%) than those with a proliferative index >3% (1 of 9, 11.1%, *p* = .025, Table 3). A lower number of tumor infiltrating CD68+ macrophages was associated with resistance to post‐surgery treatment with SRLs (48/HPFs, IQR: 22.9) than in patients responsive to post‐surgical SRL treatment (80/HPFs, IQR: 51, *p* = .005, Table 3).

### 3.5 Multivariable logistic regression

**FIGURE 5:**
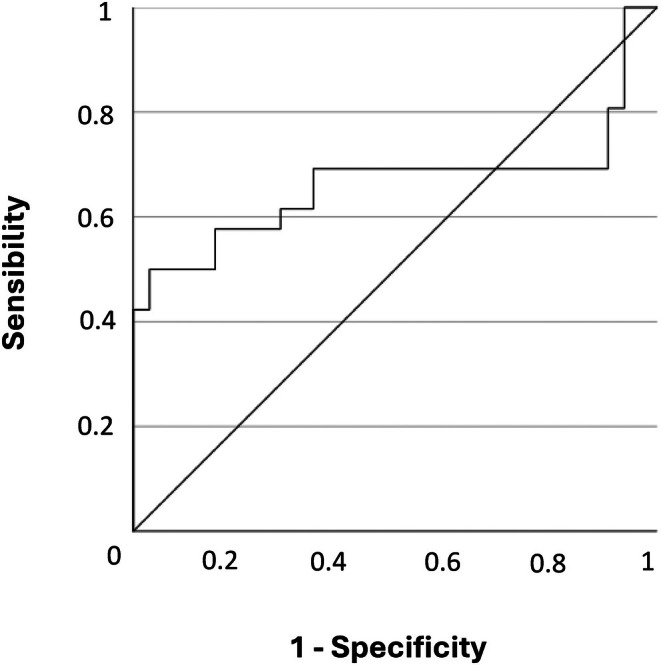
ROC Curve of CD68+/CD8+ ratio according to outcome of post‐surgery treatment with fg‐SRLs among patients naive to treatment with fg‐SRLs before surgery.

As reported in Table 4, among patients naïve to medical therapy with fg‐SRLs at surgery, independent risk factors for the occurrence of post‐surgical SRL resistance were the adenoma invasive pattern (OR: 10.5, 95% IC: 3–35.9, *p* = .05), the absent or cytoplasmic expression of the SSTR2a (OR: 2, 95% IC: 1.2–3.4, *p* = .022), and a CD68+/CD8+ ratio higher than 4.7 (OR: 4.3, 95% IC: 1.4–12.9, *p* = .006). In fact, a CD68+/CD8+ ratio lower than 4.7 (specificity: 69.2%, sensitivity: 66%, AUC: 0.663, 95% IC: 0.5–0.826, *p* = .033, Figure 5) discriminated responsive patients from those not responsive to post‐surgical SRL treatment.

**TABLE 4:** Multivariable logistic regression for predictors of response to post‐surgery treatment with fg‐SRLs in patients naïve to fg‐SRLs therapy at surgery versus patients treated with fg‐SRLs before surgery.

Covariates	OR	Naïve to SRLs therapy patients at surgery		
		95% IC	Coefficient	*p*‐value
Invasive tumors	10.5	3–35.9	0.199	.05
Sparsely granulated cytokeratin pattern	3.5	1.3–10.5	0.21	.537
Absent/cytoplasmic SSTR2a expression	2	1.2–3.4	0.113	.022
Proliferative index MIB1 >3%	3	1.1–8.4	0.37	.068
Partial surgery resection	6.1	1.9–19.9	0.15	.185
CD68+/CD8+ ratio >4.7	4.3	1.4–12.9	0.06	.028

Covariates	OR	Pre‐surgery SRLs treated patients		
		95% IC	Coefficient	*p*‐value
Proliferative index MIB1 >3%	16	1.1–234	0.11	.025
CD68+ macrophages/HFP	3	0.7–13.4	0.34	.274
CD68+/CD8+ ratio	3.2	0.4–23.5	0.28	.973

In patients who underwent presurgical SRL therapy, the only independent risk factor significantly associated with resistance to post‐surgical SRL treatment was the proliferative index >3% (OR: 16, 95% IC: 1.1–234, *p* = .025). The number of CD68+ macrophages and the CD68+/CD8+ ratio were not associated with post‐surgical SRL treatment outcome.

## 4 DISCUSSION

The immune TME has been recognized to be associated with somatotroph adenoma behavior in terms of invasive growth, proliferation, and response to SRL treatment.^14^

In this study, we compared the tumor infiltrating immune cell profile in somatotroph adenomas either naïve to SRL therapy before surgery or receiving pre‐operative SRL. For the first time, to our knowledge, this study compared somatotroph adenoma immune infiltration in patients both treated and untreated with SRL before surgery, showing significantly different immune cell infiltrates in patients naïve to presurgical SRLs and in patients who underwent presurgical SRL treatment. Interestingly, we found that the number of tumor infiltrating CD68+ macrophages and the ratio CD68+/CD8+ did not predict the outcome of post‐surgery treatment with SRL in patients that had been treated with SRLs also before surgery. This finding may therefore suggest an interplay between CD68+ macrophages and SRLs, also affirmed by reports that CD68+ macrophages express SSTRs. In a study conducted on 42 patients with atherosclerosis, gallium‐68‐labeled DOTATATE positron emission tomography (PET) was shown to be able to bind the SSTR2 on pro‐inflammatory CD68+ macrophages.^29^ Moreover, a recent study conducted on rheumatoid arthritis synoviocytes showed that octreotide suppresses production of interleukin (IL)‐15 and increases IL‐10,^30^ both components of the immune pituitary adenoma TME.^14^ IL‐10 inhibits macrophage activation and promotes their anti‐inflammatory polarization. In contrast, IL‐15 promotes maturation of macrophages and their pro‐inflammatory polarization. Therefore, supported by our results, we speculate that in pituitary adenoma, SRLs may inhibit macrophage activation through increased IL‐10. Concordantly, SRLs may reduce macrophage maturation and may suppress pro‐inflammatory activities through inhibition of the IL‐15 secretion. Consequently, the results of our study suggest that SRLs may modify the immune TME components. In fact, in patients naïve to SRLs therapy at surgery, a CD68+/CD8+ ratio >4.7 was an independent risk factor for resistance to post‐surgery/adjuvant SRL treatment.

However, it was also found that other biomarkers predict responses to post‐surgery SRL treatment in patients both naïve and in those treated before surgery with adjuvant SRLs.

Absent cytoplasmic SSTR2a expression and high MIB‐1 proliferative index of >3% were independent risk factors for resistance to post‐operative SRLs treatment only in patients naïve to SRLs at surgery. In contrast, in patients treated with pre‐operative SRLs, the number of CD68+ macrophages and the CD68+/CD8+ ratio did not correlate with the post‐surgical or adjuvant SRL responsiveness. The sole SRL‐responsiveness predictor was the proliferative index (MIB‐1).

This study therefore suggests for the first time that the treatment with SRLs may modulate the tumor inflammatory infiltrate and confirms that in somatotroph adenomas naïve to pre‐operative SRL treatment, the CD68+/CD8+ ratio was higher in invasive tumors and in those not responsive to post‐surgical SRL therapy. These findings are consistent with previous evidence showing a high number of tumor‐infiltrating CD68+ macrophages in large, invasive, and sparsely granulated somatotropinomas.^31^ This is in accordance with our previous report in a cohort of 36 patients with somatotroph adenomas naïve to medical therapy at surgery, that the number of tumor‐infiltrating CD68+ macrophages correlated with maximum adenoma diameter and was more numerous in somatotropinomas with Ki‐67 >3%.^12^ In contrast, lower numbers of tumor‐infiltrating CD8+ lymphocytes were reported in somatotroph adenomas with cavernous sinus invasion and poor response to post‐surgical SRL treatment.^12^, ^13^ In addition, a low CD8+/CD4+ ratio was identified in proliferative pituitary adenomas, with higher Ki‐67.^28^, ^32^ The CD68+/CD8+ ratio was assessed as a predictor of post‐surgical SRL responsiveness. In a cohort of 67 patients with acromegaly (naïve to medical therapy before surgery) we reported that the CD68+/CD8+ ratio was higher in somatotropinomas not responsive to post‐surgical SRLs together with the absent/cytoplasmic expression of the SSTR2A and the persistence of macroscopic adenoma tissue.^28^

The effects of pituitary adenoma therapies on TME have not been fully elucidated. Limited data are available on the effects of SRLs on TME and are mainly focused on soluble factors, such as cytokines and chemokines. In vitro studies showed that pasireotide seems to exert a more inhibitory effect on secretion of TAFs‐derived cytokines than octreotide^15^, ^33^, ^34^: in fact, pasireotide attenuates IL‐6 and CCL2 release.^15^ However, in vivo experiments did not confirm these results, as the complex network of TME and tumor cells is difficult to reproduce.^35^, ^36^ Moreover, tumor‐associated macrophages (TAMs) express SSTRs,^37^ and somatostatin has been shown to inhibit monocyte chemotaxis.^38^, ^39^ Furthermore, SRLs, particularly pasireotide, reduce expression and action of VEGF, thereby inhibiting the viability of tumor cells.^40^

The main limitations of our results are due to the lack of identification of the mechanisms by which SRLs can modulate the immune response against tumor cells. Therefore, studies in cell cultures and animal models are warranted to clarify in depth the underlying biological and molecular mechanism behind our findings. In addition, the retrospective design of our study may well associate with limitations inherent in tissue handling and processing. In this study, the number of patients treated with SRLs before surgery is limited to 33. Although the small sample of this subgroup of patients may reduce the statistical power, the results reflect our real‐life clinical practice, as in our center, the use of pre‐surgery SRLs is limited to patients with edema of the airways or obstructive sleep apnea syndrome. Therefore, prospective studies with a larger number of pretreated patients are advocated to corroborate our findings. Another limitation of this study is the application of immunohistochemistry as a single experimental model to investigate tumor immune infiltrate and the use of single markers to characterize immune cells. Moreover, the interpretation of immunohistochemistry analysis may be influenced by intra‐tumoral heterogeneity, experimental reproducibility, operator bias, experience of pathologists, and counting of tumor infiltrating immune cells. Therefore, digital analysis methods should be validated in clinical practice to standardize the analysis and interpretation of these results. However, this study reflects our clinical practice and our experimental models that were previously reported by ourselves and others. (reference) Moreover, immunofluorescence with flow cytometry and transcriptomics are also useful to investigate tumor immune microenvironment, providing superimposable results.^41^, ^42^, ^43^, ^44^ Our findings should be further investigated and validated in functional experiments to explore immune cell activity and the cytokine profiling. Future studies are advocated to fully characterize CD68+ macrophages, to differentiate M1‐polarized and M2‐polarized macrophages, and to investigate adenoma cytokines in patients naïve to SRL or pretreated with SRLs at the surgery.

Despite these limitations, our results suggest for the first time that pre‐operative SRLs may alter immune TME composition and activities. Our results shown here are consistent with prior reports^12^, ^28^ and confirm the predictive role of the CD68+/CD8+ ratio in somatotroph adenomas naïve to SRL therapy at surgery and suggest that the prognostic role of the CD68+/CD8+ ratio should be evaluated with caution in patients pretreated with SRLs before surgery.

In conclusion, our findings offer valuable insights into the interplay between somatotroph adenoma SRL therapy and the immune microenvironment, including the identification of the CD68+/CD8+ ratio as a potential biomarker for treatment resistance in SRL‐naïve patients. However, the limitations inherent in the study design and the need for functional validation warrant cautious interpretation. Future prospective studies with larger, balanced cohorts and comprehensive immunological assessments are recommended to substantiate these findings.

## AUTHOR CONTRIBUTIONS

**Sabrina Chiloiro:** Conceptualization; funding acquisition; writing – original draft; project administration; formal analysis. **Alessandra Vicari:** Investigation. **Antonella Giampietro:** Investigation. **Pier Paolo Mattogno:** Investigation. **Natalia Cappoli:** Methodology. **Greis Konini:** Investigation; writing – original draft. **Rosalinda Calandrelli:** Investigation. **Liverana Lauretti:** Investigation. **Simona Gaudino:** Investigation. **Mario Rigante:** Investigation. **Guido Rindi:** Investigation; funding acquisition; writing – review and editing. **Alessandro Olivi:** Writing – review and editing; supervision. **Laura De Marinis:** Writing – review and editing; supervision. **Antonio Bianchi:** Writing – review and editing; supervision. **Francesco Doglietto:** Writing – review and editing; supervision; project administration. **Marco Gessi:** Investigation; methodology; conceptualization. **Alfredo Pontecorvi:** Writing – review and editing.

## CONFLICT OF INTEREST STATEMENT

Sabrina Chiloiro, Antonio Bianchi, Antonella Giampietro, and Laura De Marinis have served as investigators for clinical trials funded by Novartis, Pfizer, Ipsen, and Crinetics. Sabrina Chiloiro and Antonio Bianchi received grants from Pfizer. Sabrina Chiloiro won the 2022 Arrigo Recordati Research Grant. The remaining authors declare that the research was conducted in the absence of any commercial or financial relationships that could be construed as a potential conflict of interest.

## PEER REVIEW

The peer review history for this article is available at https://www.webofscience.com/api/gateway/wos/peer-review/10.1111/jne.70078.

## ETHICS STATEMENT

All procedures performed in the study were in accordance with the ethical standards of the institutional review board and with the 1964 Helsinki declaration and its later amendments or comparable ethical standards. The study was approved by the Institutional Review Board of the University Hospital “Fondazione Policlinico Universitario A. Gemelli IRCCS” in Rome, Italy (Protocol ID: 5132). Informed consent was obtained from all subjects involved in the study.

## Supporting information


**Table S1.** Details of the primary antibodies used for immunohistochemistry.

## Data Availability

The data that support the findings of this study are available on request from the corresponding author. The data are not publicly available due to privacy or ethical restrictions.
