# Supplementary material for: Tumor‐infiltrating immune cells predict the response to somatostatin receptor ligands only in somatotropinomas naïve to medical therapy
Source: J Neuroendocrinol. 2025 Aug 11;37(11):e70078. doi: 10.1111/jne.70078 (PMC12580450; doi:10.1111/jne.70078)
Supplement: Supplementary file 1 — Table S1. Details of the primary antibodies used for immunohistochemistry. [file JNE-37-e70078-s001.docx]

| **Antibody (clone)** | **Supplier** |
| --- | --- |
| **KI67 clone MM1** | Leica Microsystems |
| **p53 clone D07** | Leica Microsystems |
| **CD138 clone MI15** | Leica Microsystems |
| **CD8 clone 4B11** | Leica Microsystems |
| **CD68 clone 514H12** | Leica Microsystems |
| **SSTR2A clone UMB1** | Abcam Dako Omnis Dab Low |
| **PIT1 clone nbp1-92273** | Novus Ultra View |
| **GATA3 clone L50-823** | Roche Diagnostics |
| **Cytokeratin 8/18 clone 5D3** | Leica Microsystems |

Table 1. Details of the primary antibodies used for immunohistochemistry.
